# Realguard: A Lightweight Network Intrusion Detection System for IoT Gateways

**DOI:** 10.3390/s22020432

**Published:** 2022-01-07

**Authors:** Xuan-Ha Nguyen, Xuan-Duong Nguyen, Hoang-Hai Huynh, Kim-Hung Le

**Affiliations:** 1 Faculty of Computer Networks and Communications, University of Information Technology, Ho Chi Minh City 70000, Vietnam; 18520042@gm.uit.edu.vn (X.-H.N.); 18520697@gm.uit.edu.vn (H.-H.H.); 2 Faculty of Computer Science, University of Information Technology, Ho Chi Minh City 70000, Vietnam; 18520212@gm.uit.edu.vn; 3 Vietnam National University, Ho Chi Minh City 70000, Vietnam

**Keywords:** network intrusion detection system, deep neural network, IoT gateways

## Abstract

Cyber security has become increasingly challenging due to the proliferation of the Internet of things (IoT), where a massive number of tiny, smart devices push trillion bytes of data to the Internet. However, these devices possess various security flaws resulting from the lack of defense mechanisms and hardware security support, therefore making them vulnerable to cyber attacks. In addition, IoT gateways provide very limited security features to detect such threats, especially the absence of intrusion detection methods powered by deep learning. Indeed, deep learning models require high computational power that exceeds the capacity of these gateways. In this paper, we introduce Realguard, an DNN-based network intrusion detection system (NIDS) directly operated on local gateways to protect IoT devices within the network. The superiority of our proposal is that it can accurately detect multiple cyber attacks in real time with a small computational footprint. This is achieved by a lightweight feature extraction mechanism and an efficient attack detection model powered by deep neural networks. Our evaluations on practical datasets indicate that Realguard could detect ten types of attacks (e.g., port scan, Botnet, and FTP-Patator) in real time with an average accuracy of 99.57%, whereas the best of our competitors is 98.85%. Furthermore, our proposal effectively operates on resource-constraint gateways (Raspberry PI) at a high packet processing rate reported about 10.600 packets per second.

## 1. Introduction

Recent years have seen the proliferation of the Internet of Things (IoT) and its impact on various domains from agriculture, healthcare, transportation to automotive industry. Aiming to bring every physical object into digital worlds, IoT connected billions of devices, which are embedded with sensors, actuators, and other technologies, to the Internet and generated zillions bytes of data. According to [1], 75 billion IoT devices are deployed by 2025 and expected to generate 79.4 zettabytes (ZB) of data. By deeply analyzing these data, IoT providers offer various intelligent services, such as adaptive watering in smart agriculture, predictive maintenance in smart factories [2]. These services not only boost business profits but also enhance user experience. Fortune Business Insights reported that business for IoT could reach 1463.19 billion dollars by 2027 and a compound annual growth rate of 24.9% [3]. In summary, the Internet of Things is recognized as a key element of the digital revolution in reshaping our society.

However, cyber-security and data privacy risks are the top concerns for fully unleashing IoT benefits [4]. An IoT system comprises numerous smart devices (e.g., sensors, actuators) which have limited computational resources and heterogeneous hardware. Employing complex and efficient security countermeasures on such devices is highly nontrivial and may affect device performance or even damage the devices. This causes a substantial gap between security requirements and the security capability of existing IoT devices. Therefore, these devices is vulnerable to a wide range of cyber attacks from the Internet, such as spoofing, distributed denial-of-service (DDoS) [5]. Several intensive cyber attacks targeting IoT devices are recorded, leading to undesirable effects, such as damaging device hardware, disrupting the IoT system. For example, the Mirai malware performed the most famous IoT attack in 2017, infecting over 380.000 IoT devices and turning these devices into malicious botnets to perform DDoS attacks. According to US-CERT, this is the most significant DDOS attack recorded [6]. Another attack targeting steel factories in Germany compromised the controlling system of blast furnaces, giving rise to potential blocks to supervision functions (e.g., automated stop in emergent cases) [7]. This makes the entire production line delayed, resulting in enormous damage to manufacturing productivity. Similarly, cyber attacks in the Ukraine power grid and smart home system in London are also typical examples of significant IoT security risks [8].

To mitigate the security risks of IoT systems, a network intrusion detection system is commonly deployed at an Internet gateway to secure the networks [9]. It continuously monitors all network traffic and detects potential signs of malicious activities. However, the NIDS inspects only inbound and outbound traffic, but not internal traffic. To solve this issue, a distributed deployment strategy, in which the NIDSs are integrated into routers and gateways within the networks, is demanded [10]. Because of moving near network traffic sources (IoT devices), it also reduces network traffic volume and complexity, resulting in increasing the detection accuracy of these NIDS. On the other hand, motivated by the success of machine learning in several fields (e.g., computer vision, robotics, automation), several groups in the scientific and commercial communities focus on leveraging machine learning models to improve the NIDS detection performance [11]. One popular approach is employing a deep neural network (DNN) to classify network traffic into normal and abnormal classes [12]. This DNN model is first trained by labelled datasets containing both normal and attack traffic before deploying NIDS to detect cyber threats. Compared with traditional machine learning methods, DNN can better learn complex non-linear features being common in network traffic data [13].

The work presented in this paper is motivated by the fact that: Embedding a DNN-based NIDS into typical gateways of IoT system is highly nontrivial. Indeed, DNN models require high computational power that exceeds the capacity of these gateways [14,15,16,17]. The main technical goal is thus to provide a lightweight DNN-based NIDS suitable for resource-constrained IoT gateways. This means that the proposed NIDS could be deployed and executed on IoT gateways with minor resource consumption (CPU and RAM). By achieving this goal, we could offer a more secure and resilient IoT network architecture. For instance, collected data from IoT devices are transferred to a cloud server through a gateway with unreliable connection. In this scenario, this gateway appears vulnerable to network attacks, so embedding an effective NIDS directly into it is essential.

In this paper, we designed, implemented, and evaluated a DNN-based NIDS named Realguard that can detect both internal and external cyber-threats effectively. In more detail, we focus on the following objectives:

- Offer better protection for IoT devices by moving the NIDS to the IoT network gateway. Indeed, moving near network traffic sources (IoT devices) could not only increase NIDS’s detection accuracy by lowering the volume and complexity of incoming network traffic, but also rapidly identify both internal and external cyber threats. To do this, the proposed NIDS must be lightweight enough to operate on resource-constrained devices while ensuring sufficient detection performance. In addition, it demands operating in real time to satisfy latency requirements. This means that the packet processing rate of the NIDS must be higher than the estimated packet arrival rate of the IoT network to guarantee there is no waiting packet.
- Identify multiple cyber-attack, including ensemble attacks merged from several attack strategies. In more detail, the proposed NIDS has to identify a large set of attacks from malicious signs in the network traffic. To achieve this aim, we proposed a DNN model that effectively detects ten popular attacks in the IoT domain with high accuracy. We note that executing the model must consume minor computational resources to make it suitable for the former objective.

The main contributions of this work can be summarized as:

- We present Realguard, a DNN-based intrusion detection system operated directly on an IoT edge gateway. The proposed NIDS not only automatically distinguishes between normal and abnormal network traffic but also effectively identifies the various attacks from external attackers or internal compromised devices. It is interesting to note that Realguard has a small memory footprint and high packet processing rate because of the superiority of our DNN model acting as an anomaly detection algorithm. To the best of our knowledge, we are the first to propose the use of a DNN-based NIDS on resource-constraint IoT gateway in real time.
- We propose an efficient feature extraction module to extract network features from network traffic. To increase extraction speed, we also applied a Damped Incremental Statistic algorithm that boosts the extraction speed on concurrent data streams, and the time complexity is only O(1).
- We demonstrate that Realguard can fully operate on resource-constrained IoT gateways, while detecting a wide range of cipher threats (10 attack types) in real time with a very low false-positive rate. To evaluate the packet processing rate of our proposal, we implemented a labeling method to convert the CICIDS2017 dataset into a packet-based dataset that could be reused by the research community.
- We pack all the components of our proposal into a Docker container which is publicly available for the research and development of open communities. This container can be seamlessly integrated into any IoT gateway frameworks supporting Docker containers (e.g., AGILE [18], Balena [19]). In addition, the integration process could be automated by using Jenkins [20], an open-source automation server. This promotes the popularity of our proposed system and makes it become platform-independent.

The rest of this paper is presented as follows: Section 2 introduces some approaches related to deep learning-based NIDSs. Section 3 details our proposed model design. Section 4 presents our experiment implementation, dataset, evaluation metrics, and our experiment results. Finally, Section 6 show paper summarizing and recommending possible research topics.

## 2. Related Works

To find our related works, we included the studies in the last five years that (1) detect cyber attacks using machine learning-based intrusion detection systems; (2) comprehensively evaluate the detection performance on well-known IDS datasets. These studies are found on several academic research databases (e.g., ACM digital library, scopus, ieee xplore) through various query strings, such as “deep learning-based NIDS”, “machine learning-based NIDS”, “IDS for IoT”. 281 studies in total are found and skimmed; finally, 24 studies are reported in this section and the most significant research works are summarized in Table 1.

Motivated by the success of machine learning, several research groups have been working on integrating machine learning into NIDS to enhance attack detection quality. Ref. [30] presented the literature on machine learning technologies used in cybersecurity during the last decade, including intrusion detection, spam detection, and malware detection on computers and mobile networks. It also provided an overview of the issues encountered by machine learning technology. The authors in [31,32] assess the efficacy of several ML models on IDS datasets. From the evaluation results, they discussed the obstacles and constraints in employing machine learning in cybersecurity. Ahmin et al. presented a two-stage NIDS that incorporates several classification methods, including REP Tree, JRip algorithm, and Forest PA algorithm [24]. The attack detection process is separated into two stages. The first stage employs the two first classifiers to identify whether the network traffic is benign or malicious, and the second stage employs the third classifier to identify the attack types. In the experiments on the CICIDS2017 dataset, the proposed IDS could achieve accuracy rate of 96.66%. Liu et al. introduced a CNN-based NIDS model which achieves 97.7% accuracy on the KDD’Cup99 data set and outperforms traditional machine learning algorithms (such as KNN, SVM) [22]. In [33], the authors adopted a LeNet-5 based Convolutional Neural Network (CNN) model to classify attacks. To increase the detection quality, they also applied the information gain scheme to reduce the number of features. The experiments on 10,000 samples of the KDD’Cup99 data set show that their model could detect 97.5% abnormal traffic. Faker et al. implemented a distributed scheme for NIDS combining with a DNN and two ensemble techniques: Random Forest and Gradient Boosting Tree (GBT) [25]. They developed a prototype using Apache Spark and evaluated it using the 5-fold cross-validation technique on UNSW NB15 and CICIDS2017 datasets.

In [27], the authors presented a hybrid NIDS model combining CNN and LSTM to enhance the performance of an IDS. In addition, they optimized the model training phase using category weights, resulting in decreasing the number of unbalanced samples in the training dataset. The proposed NIDS is validated on the CICIDS2017 dataset, which contains seven types of network traffic, and achieves 98.67% accuracy with a false alarm rate recorded about 0.47%. However, this NIDS is ineffective in detecting the Heartbleed and SSH-Patator attacks. To overcome this issue, Kaiyuan et al. developed a NIDS method that incorporates hybrid sampling and a deep hierarchical network [29]. They also employed one-side selection to handle noise on minority labels and the synthetic minority over-sampling technique to expand the sample size of minority labels. This may enhance the detection performance on imbalanced datasets and decrease training time. In more detail, after preprocessing the network features, they extracted spatial characteristics using CNN and temporal information using bi-directional long short-term memory. Therefore, the experimental results of the NSL-KDD and UNSW-NB15 datasets show that their proposed method outperforms existing works.

In [34], the authors demonstrated the potential of deploying NIDS down to the IoT edge network. They attempted to install two machine learning models, Isolation Forest (iForest) and Local Outlier Factor (LOF), on resource-constrained devices to identify network attacks. In the experiments, the model is capable of classifying four types of attacks and consuming small computation resources. Midi et al. developed Kalis, a network monitoring and configuration tool for IDS based on network-specific detection techniques [21]. Kalis employs both signature-based and anomaly-based techniques to detect malicious traffic. Additionally, it gathers data from network modules to avoid DoS attacks using traffic analysis, current information, and network topology. The major limitations of Kalis are the routing assaults and demanding specialized detection modules. Those cause complexity to the network and may result in suboptimal detection performance.

Mirsky et al. developed a system called KitNet that applies autoencoders to identify security threats [23]. They also presented an efficient feature extraction method to speed up Kitnet’s processing rate. The experiments show that the KitNet could achieve 94.47% accuracy and process 37,300 packets per second. However, these experiments are only performed on self-generated data that contains limited network attack types. Similarly, the authors in [26] developed a deep hierarchical model to detect abnormal network traffic at the packet level. The model is based on a mix of CNN and Gated Recurrent Units (GRU); it is evaluated on three data sets: ISCX2012, USTC-TFC2016, and CICIDS2017, and achieves 99% accuracy. Additionally, its processing rate is reported at about 20,000 packets per second.

In [35], the authors presented and analyzed existing security issues in the IoT context. They also reviewed the proposed solutions for these issues and discussed open challenges. The authors in [36] addressed security challenges for edge computing systems, including limited computational capability and high data volume collected from end devices. They also suggested a design of architecture to detect cyber-attack on low-cost devices. The evaluation results demonstrated that the model works effectively with a small memory footprint. In [37], the authors modeled the operation behavior of IoT network events using CoAP protocol and automated controller and applied it to a Hybrid NIDS model to minimize energy usage while retaining accuracy up to 99.17%. Ref. [38] proposed a hierarchical approach to identify selective forwarding attacks on wireless sensor networks. The proposed method combines machine learning techniques to improve detection accuracy to more than 95%. In [34], the authors developed an intelligent Intrusion Detection System (IDS) named Passban to safeguard the IoT edge network. The proposed approach is lightweight enough to operate on IoT gateways. The evaluation results on real-life scenarios demonstrated that Passban installed in a cheap IoT gateway board could detect several cyberattacks with small resource consumption. Other research has also expanded their investigation into cyber security’s sub-domains. For example, the work in [39] investigated the features of several aspects in time-series anomaly detection. Ref. [40] provided a mutual authentication scheme with the outstanding feature of minimal complexity and a simple setup to install for resource-constrained devices.

Although deep learning algorithms have made remarkable accomplishments, problems are still identified with privacy and security issues when the DL model can be stolen, reversed, or poisoned by adversarial attacks. In a security-related study for DL models, the authors analyzed four common types of attacks focusing on DL algorithms, including model extraction attacks, model inversion attacks, adversarial attacks, and poisoning attacks [41]. To counter these kinds of attacks, the authors also review existing privacy-preserving technologies, including cryptography, the trusted execution environment, and digital watermark. The study provides the analysis, compares the cons and pros and the effectiveness of the above techniques, then finishes with the open challenges. In [42], the author has developed a solution to address the resource needs of efficient service in the industrial IoT called BrainIoT. A resource reservation algorithm is proposed in the Industrial IoT using federated learning to optimize resources between network connections. Ref. [43] proposed an efficient data sharing and searching scheme to enable access control and data sharing securely among edge devices in a cloud-assisted IoT system. The authors analyzed the performance and proved that their approach is practical and suitable for IoT applications. In addition, Ref. [44] explored two defense techniques that can help safeguard intelligent vehicles from cyberattacks: blockchain and machine learning. It has facilitated the development of a defense technique, which is resource-saving, lightweight, and efficient, for smart devices in cybersecurity.

## 3. The Realguard IDS

In this section, we present the Realguard NIDS, including the architecture overview, the feature extraction, and the attack detection model.

### 3.1. Overview

**Figure 1:**
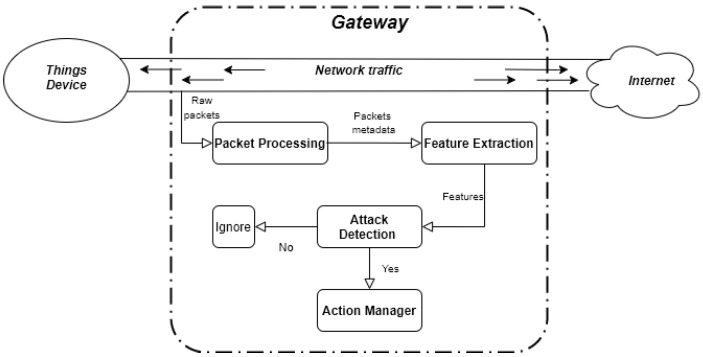
The workflow of the Realguard IDS.

Realguard is a deep learning-based NIDS, designed to accurately detect a wide range of cyber attacks in network traffic. Its operation includes (1) monitoring and extracting statistical features from network traffic, and (2) detecting attack signs using a DNN model. To run on resource-constrained IoT gateways, Realguard has been designed with low computation footprint (CPU and RAM) while ensuring high attack detection performance. To illustrate how it works, Figure 1 provides the overview of the proposed IDS, including the data flow direction and the interfaces among components of the IDS. In more detail, Realguard is composed of four main components:

- **Packet Observation Component (POC)**: It continuously captures network traffic passing gateway and extracting their metadata information. There are several open-source tools that could perform this task, such as NFQueue [45], afpacket [46], and tshark [47].
- **Feature Extraction Component (FEC)**: It calculates the network traffic statistic based on the collection of packet metadata in previous blocks. The statistic is then formed as feature vectors compatible with the deployed detection model. In more detail, *n* features are extracted from network packets to create a network vector $\bar{x}\in R^{n}$. To increase the extraction rate, we applied the Damped Incremental Statistics algorithm [23].
- **Attack Detection Component (ADC)**: It is responsible to detect abnormality in network traffic in real-time given network features. To fulfill this aim, we proposed a DNN model that not only detects whether an attack occurred or not, but also identifies the attack type. This ability is important for system administrators to quickly deploy appropriate countermeasure methods to prevent or reduce the severity of the attack.
- **Action Manager Component (AMC)**: It is responsible to provide necessary actions based on pre-configuration from users when an attack is detected by ADM. In Realguard, the supported actions are to log and block abnormal traffic, send the notification to administrators by email.

The network feature extraction and attack detection are the most essential and complex components of a NIDS. Thus, to operate on resource-constrained devices, the complexity of these components is demanded to reduce while maintaining high accuracy. In Realguard, we present several features to solve this challenge:

- In the feature extraction component, an exponential decay function is used to calculate the weight of network packets that is exponentially decreased over time. The packet information is removed to save memory if its weight equals zero. In addition, instead of storing the statistical values of packets in a sliding window, which has O(n) complexity, the cumulative sum is employed to accumulate these values, decreasing the complexity to O(1).
- In the attack detection component, a DDN model consisting of five hidden layers with only 34,315 parameters is proposed and produces a high detection performance while ensuring simplicity.

To have a better insight in understanding of how Realguard works, we now illustrate the block diagram showing the operation process of Realguard in Figure 1.

1. The **POC** monitors and captures inbound and outbound network packets that are used to extract metadata information relating network traffic statistic (e.g., IP, port, packet size, timestamp, protocol, and so on). The metadata information is then sent to **FEC** for further processing.
2. The **FEC** computes numerous statistical features to describe the current network states from the received information. Due to the variety of IoT devices (considering as data sources in the network) and network topologies, the value of extracted features is highly diverse, so these features are normalized and formed into a vector $\vec{v}$ before being transferred to **ADC**.
3. The Component receives vector $\vec{v}$ and uses it differently in two modes:
  - **In training mode**: We group these vectors according to their respective labels, and then divide them into two subsets of data used for training and validating. After training the model is complete, we receive an output model and deploy this model on network devices under executing mode.
  - **In executing mode**: The model executes $\vec{v}$ and returns an integer number indicating the type of the network packet. In Realguard, we could classify 10 attack types. If any attacks are detected, a notification command is sent to **AMC**.

Since Packet Observation Component and Action Manager Component are not our principal contribution and well-described in several public documentations, we mainly discuss how the Feature Extraction Component and Attack Detection Component work in greater detail.

### 3.2. Feature Extraction Component

In the network security context, feature extraction refers to a procedure that employs one or more methods to derive network attributes from network packets. These attributes reflect the current network activities and may be used to identify abnormal behaviors in the network. For example, relying on the rate of TCP SYN packet, we could recognize a DoS attack in our network if such rate is significantly higher than usual. Similarly, the sudden emergence of SYN packets in jitter may indicate a man-in-the-middle attack. Therefore, extracting appropriate network features is instrumental in detecting cyber threats. However, a feature extraction method for IoT network traffic faces several challenges because:

- An IoT network contains several devices that may create a large number of parallel sessions. This makes extracting valuable information about the relationships between these sessions more challenging.
- The network packets observed from simultaneous sessions are often intertwined with each other.
- Since the network sessions have different duration and traffic volumes, the feature extraction may consume a large amount of memory to store the session information.
- The network packet rate is enormous under DoS/DDoS attacks, up to millions of packets per second.

To address these challenges, we designed a feature extractor based on the Damped Incremental Statistics algorithm. This algorithm comprises different techniques that can remedy the above challenges: (1) running total technique, which updates the total values each time by adding the new value to the previous one, gathers the information from packets on multiple sessions in order; (2) An exponential decay function is used to calculate the weight of network packets that is exponentially decreased over time. The packet information is removed to save memory if its weight equals zero. (3) Instead of storing the statistical values of packets in a sliding window, which has O(n) complexity, the cumulative sum is employed to accumulate these values, decreasing the complexity to O(1). Our proposal could concurrently extract about 100 network features on different data streams at high speed. Briefly, the proposed method is as follows:

Let $V=x_{1},x_{2},\ldots$ ($x_{i}$ ∈ *R*) is an unbounded data stream, and the decay function $d_{\alpha }\left(t\right)$ is defined as:

(1) $$d_{\alpha }\left(t\right)=2^{-\alpha t}$$

where α is decay factor and *t* is the timestamp difference between two observation.

For each stream $V_{i,\alpha }$, the feature extractor maintains an array $IV_{i,\alpha }$ containing the current weight *w*, the sum of residual products between two attribute streams $SR_{ij}$, the last updated timestamp of the array $t_{L}$, the linear sum of all instances LSI, and the squared sum of all instances SSI.

(2) $$IV_{i,\alpha }:=\left(w,LSI,SSI,SR_{ij},t_{L}\right)$$

To update the array $IV_{i,\alpha }$ in realtime with $x_{C}$ at time $t_{C}$, the feature extractor follows the update steps illustrated in Algorithm 1.
**Algorithm 1:** Update $IV_{i,\alpha }$**Input   **: $IV_{i,\alpha },x_{C},t_{C},r_{j}$**Output**: $IV_{i,\alpha }$1$\delta \leftarrow d_{\alpha }\left(t_{C}-t_{L}\right)$2$IV_{i,\alpha }\leftarrow \left(\delta w,\delta LSI,\delta SSI,\delta SR_{ij},t_{C}\right)$3$IV_{i,\alpha }\leftarrow \left(w+1,LSI+x_{C},SSI+x_{i}^{2},SR_{ij}+r_{i}r_{j},t_{C}\right)$4return $IV_{i,\alpha }$

Because of the wide variety of network activities, the values of extracted features are highly variable. Thus, these values must be normalized before further processing. Let $x\in R^{n}$ denote the set of extracted features with n=100. The normalization value of *x* is defined as:

(3) $$z_{i}=\frac{x_{i}-\mu_{i}}{s_{i}}\left(\forall i:1\leq i\leq n\right)$$

where μ and *s* are the mean and the standard deviation of feature *i*th (1≤i≤n).

### 3.3. Attack Detection Component

Over the past decade, there has been a sustained research activity in applying machine learning techniques (e.g., CNN [48], LSTM [49], and traditional machine learning algorithms [50]) to detect cyber attacks. However, these techniques still have their own limitations. For example, CNN is good at extracting the information in the adjacent values that are rarely available in network traffic data. Traditional machine learning algorithms are low-complexity, but they are inefficient in handling the complex relationships of network traffic. Therefore, the DNN model is a potential candidate anticipated to detect cyber attacks accurately with a low resource footprint. In Realguard, we designed the attack detection component based on a lightweight DNN model that is capable of inferring the non-linear and complex relationship between network features and the attack signs.

**Figure 2:**
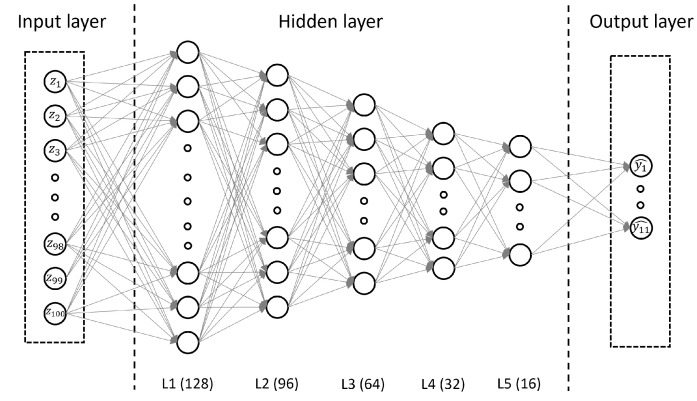
The architecture of the attack detection model.

One of the most significant challenges we faced is how to optimize the number of hidden nodes in the network to achieve Realgual’s objectives, which is to accurately detect attacks with low resource consumption. A large number of hidden nodes could increase model accuracy but lower detection speed and lead to over-fitting. This challenge is more complex when working with discrete datasets (e.g., traffic network datasets) because the model architecture must be sufficient depth and wide to create the connections between model inputs. To solve it, we employed the growing approach that starts with a minimal network, which only has input nodes and the output nodes, and then inserts hidden layers and hidden neurons into the network until satisfying our criterion [51]. The optimized model we proposed includes five hidden layers and about 34,315 parameters in total, producing a high detection performance while ensuring simplicity. As shown in Figure 2, each of the hidden layer is constituted by neurons, each of which is fully connected to all neurons in the next layer. The information is transformed from one layer to another in a forward direction with neurons in each layer. In more detail, the input layer, consisting of *n* neurons, receives normalized vector $z\in R^{n}$ (n=100) from the feature extractor as a parameter. Let $d_{i}$ is the output size of layer $L_{i}$, the computation of each hidden layer $L_{i}$, input as a vector $x\in R^{d_{i-1}}$ which is the output of layer $L_{i-1}$ or the input vector *z*, is defined as:

(4) $$L_{i}\left(x\right)=f\left(w_{i}^{T}x+b_{i}\right)$$

where $f:R^{d_{i-1}}\to R^{d_{i}}$ is the activation function, $w_{i}\in R^{d_{i-1}\times d_{i}}$ is a weight coefficient matrix, and $b_{i}\in R^{d_{i}}$ is the bias vector. In other words, the operation $w_{i}^{T}x+b_{i}$ maps values from layer $L_{i-1}$ to layer $L_{i}$. For the activation function *f*, we use an advanced non-linear function, namely ReLU, to reduce the likelihood of vanishing gradient and gain better convergence performance than other linear functions [52]. Let $x^{r}=w_{i}^{T}x+b_{i}$, the element *j*th of vector $L_{i}\left(x\right)$ is computed by the following formula:

(5) $$f\left(x_{j}^{r}\right)=\max\left(0,x_{j}^{r}\right)$$

Additionally, our architecture requires categorization into one or more than two attack types, so we need a tool to determine the type of attack to which a received input belongs. Softmax function is widely used for this sort of task. By reducing the size of the final layer to the same as the number of attack types. The softmax function could compute the probability of a sample belonging to classes, which can be simply expressed by

(6) $$\hat{y_{k}}=\frac{e^{x_{i}}}{\sum_{j=1}^{m}e^{x_{i}}}$$

where $\hat{y_{k}}$ is the probability varying from 0 to 1 of a received input represented by vector *x* belongs to *k*th attack and *m* is the number of attack types.

To optimize the weights and biases of the model, we employ stochastic gradient descent (SGD) algorithm, a variation of backpropagation. The SGD algorithm computes the mathematical distance, namely categorical cross-entropy loss, is defined by the following formula:

(7) $$L=-\frac{1}{N}\sum_{i=1}^{N}\sum_{k=1}^{m}y_{k}^{\left(i\right)}\log\left(\hat{y_{k}^{\left(i\right)}}\right)$$

where *y* is a one-hot vector of the labels, and *N* stands for the number of training dataset samples. Using the chain rules, it adjusts the parameter values based on the gradient of *L* value.

## 4. Evaluation

Realguard is a DNN-based intrusion detection system operating directly on IoT edge gateways with a small memory footprint and high packet processing rate. It not only automatically distinguishes between normal and abnormal network traffic, but also effectively identifies the various attacks from external attackers or internal compromised devices. In this section, we provide an evaluation of Realguard about its detection and runtime performance that is separated into two parts. The first part describes the evaluated datasets and evaluations metrics. The second part presents evaluation results in detail, including binary and multi-class attack detection performance, baseline comparison, and runtime performance. Note that we analyzed several state-of-the-art machine learning based intrusion detection system as our competitor, including XGB [53], AE+ANN, RF, LSTM [54], PSO-LSTM-RNN and PSO-DNN [55], DBN [56], NB-SVM, DT-EnSVM [57], E-ML and REP-Tree [24], and CNN-MCL [28],

### 4.1. Evaluation Environments

Realguard aims at providing a lightweight IDS that could adopt at edge gateways to detect multiple attacks in real-time. Given this aim, we evaluated our proposal on two different environments:

- Edge gateway is represented by a single board computer (Raspberry PI 4B), which has Quad core Cortex-A72 processor and 8GB RAM.
- Edge server is represented by a PC, which is equipped with 8 Intel-i7 processors and 16 GB available memory.

All environments run on a 64-bit Ubuntu operating system. They are also installed Keras version 2.4.3 and Tensorflow version 2.4.0 to handle the attack detection model. The details of evaluation environments are described in Table 2.

### 4.2. Datasets

In our experiment, the CIC-IDS2017 dataset is employed to evaluate the detection and runtime performance of Realguard. This dataset published by the University of New Brunswick contains a sufficient amount of abnormal network traffic from common cyber attacks to train deep learning models [58]. The network traffic was collected from 9 a.m. on 3 July 2017 to 5 p.m. on 7 July 2017, producing 51.1 Gigabytes data and 13 types of network traffic (benign and 12 attack types). Furthermore, this dataset provided raw network traffic data in pcap format, which allows us to assign labels to each packet rather than using Flow ID to evaluate the processing rate of our proposal. As shown in Table 3, the packet-labeled dataset has approximately 15,950 million labeled packets in the dataset, with approximately 12 million normal packets. In order to address the imbalance in this dataset while meeting multi-classification criteria, 200,000 packets for each attack type and 400,000 normal packets are chosen for experiments by using the K-fold cross-valuation method with k=5.

### 4.3. Evaluation Metrics

To measure the detection performance of the attack detection model, we employ four common machine learning evaluation metrics including:

- Confusion matrix: It is a specific table with two rows and two columns that present the values of true positives (TP), true negatives (TN), false positives (FP), and false negatives (FN).
- True Positive Rate (TPR or Recall or Detection Rate): It is the ratio of abnormal activities correctly detected over the total of abnormal activities.
  $$\begin{array}{c} \mathrm{TPR}=\frac{\mathrm{TP}}{\mathrm{TP}+\mathrm{FN}} \end{array}$$
- False positive rate (FPR or Fallout or Fall Alert): It is the rate of abnormal activities imprecisely detected over the total of normal activities, also known as the false alert rate.
  $$\begin{array}{c} \mathrm{FPR}=\frac{\mathrm{FP}}{\mathrm{FP}+\mathrm{TN}} \end{array}$$
- Accuracy (ACC): It is the ratio of accurately detected activities over all activities.
  $$\begin{array}{c} \mathrm{ACC}=\frac{\mathrm{TP}+\mathrm{TN}}{\mathrm{TP}+\mathrm{FP}+\mathrm{TN}+\mathrm{FN}} \end{array}$$
- Confusion matrix: It is a table widely used to visualize the performance of classification method. Its rows represent the actual classes, while its columns represent the predicted classes. In our evaluation results, each cell of the confusion matrix presents the number of correct predictions and the TPR values.

### 4.4. Results and Discussion

**Figure 3:**
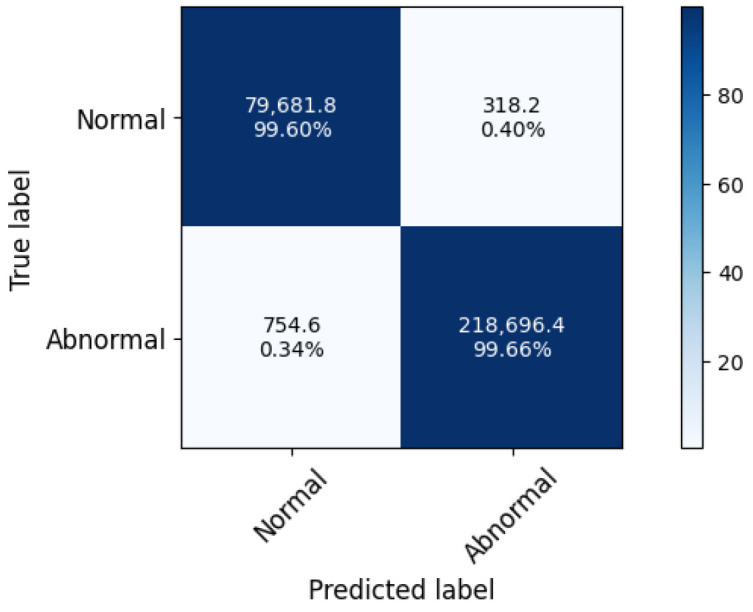
The experiment results of the binary-class attack detection.

**Binary-class attack detection:** We first assess the detection quality of our proposal about differentiating between normal and abnormal network traffic. As shown in Figure 3, our TPR and TNR values are reported about 99.6% and 99.66%, respectively. This means that Realguard could accurately identify malicious network traffic due to the superiority of the proposed attack detection model. Moreover, a comparative analysis of detection quality in Table 4 shows that Realguard outperforms its competitors in correctly detecting abnormal activities in network traffic although its fall alert rate is slightly higher than the best of competitors. For example, the accuracy and FPR of Realguard are 99.64% and 0.4%, whereas the best ones of the competitors are reported about 98.92% and 0.16%.

**Figure 4:**
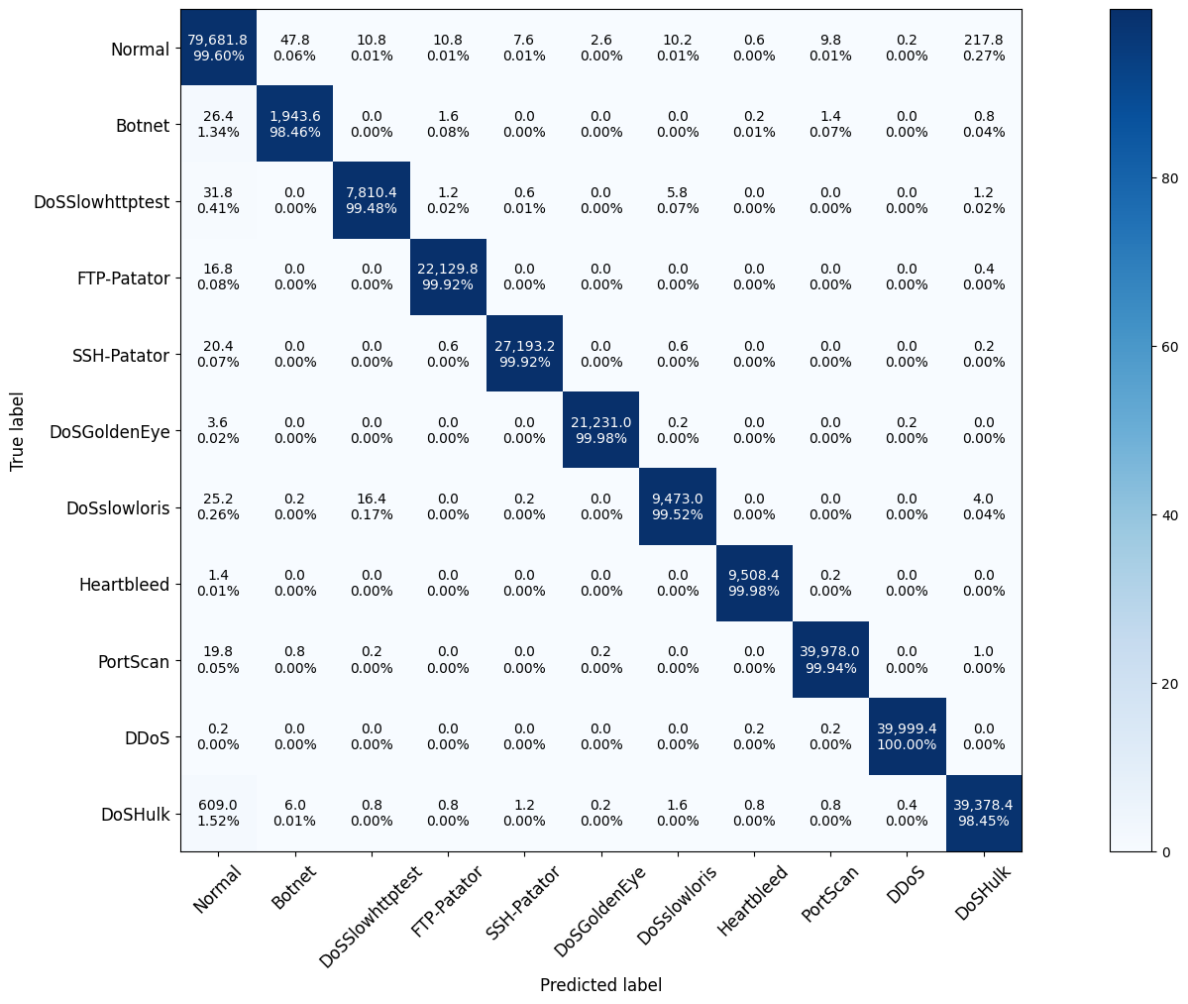
The experiment results of the multi-class classification.

**Multi-class attack detection: **Figure 4 presents the detection performance of Realguard for each type of attack. We can easily see that Realguard correctly identify 10 attack types with an average TPR measured about 99.57%. The lowest TPRs are recorded at the Dos Hulk and Botnet attacks (about 98.45%). Deeper investigating attacks, we found that 1.52% of Dos Hulk and 1.34% of Botnet samples are miss-classified into normal since these samples were evenly distributed throughout the attack duration. Indeed, in the Dos Hulk attack, the attackers consistently send valid packets to the target, and our attack detection model considers these packets belonging to normal activities.

**Figure 5:**
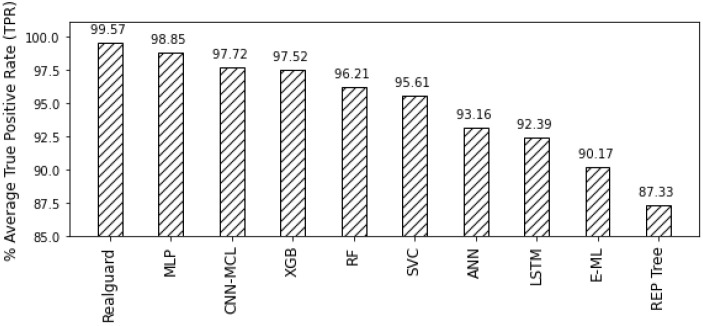
Comparing the TPR value of the multi-class attack detection between Realguard and its competitors.

**Figure 6:**
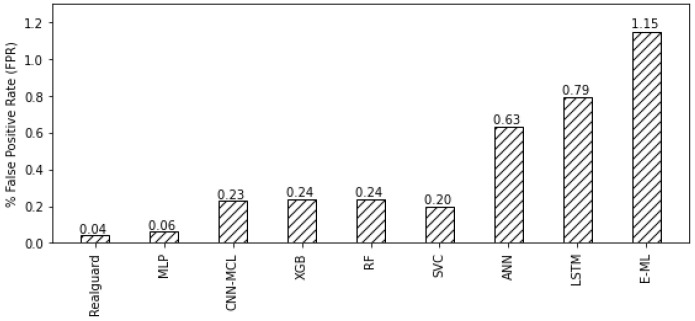
Comparing the FPR value of the multi-class attack detection between Realguard and its competitors.

For a baseline comparison, we first compare the TPR and FPR values of Realguard with competitors and subsequently illustrate the results in Figure 5 and Figure 6. As shown in these figures, it is clear that our proposal outperforms competitors in terms of TPR and FPR. Indeed, Realguard achieves the highest TPR while maintaining the lowest FPR compared with competitors. Its TPR and FPR values are 99.57% and 0.04%, whereas the best of others is 98.85% and 0.04%, respectively. A more detailed comparison in Table 5. We note that the colored box shows the best values in rows. Interestingly, although Realguard only has the highest TPR values at the three attacks (“BotNnet”, “DoSGoldenEye” and “DDoS”), all its evaluation metrics (TPR, FPR, and accuracy) are higher than ones of its competitors. This results from the low-quality variation in detecting different cyber attacks. In summary, the evaluation results illustrate the superiority of Realguard over existing IDS solutions in detecting multiple security threats.

**Runtime performance:** The superiority of Realguard is to detect cyber attacks in real time with low resource consumption, which is suitable for IoT gateways. To prove this advantage, we evaluate the training and processing rates of our proposal in the evaluation environments (PC and Raspberry Pi) and compare it with recent real-time IDS named Kitsune [23]. As shown in Table 6, Realguard outperforms its competitor in both environments. On PC, our training and processing rates are recorded about 6000 packets per second (packet/s) and 88.200 packet/s, whereas the ones of Kitsune are 1100 packet/s and 37.300 packet/s, respectively. Similar results are also found on Rasberry Pi, where our processing rate is doubly faster than the rate of Kitsune. In addition, our attack detection model can be trained on edge gateways with the training rate measured at 1150 packet/s. We also record the memory footprint and CPU usage of running Realguard and its competitors on Rasberry and present in Table 7. As can be seen from the table, our proposed IDS consumes low computational resources, only 114.5 MB RAM and 36% CPU, and is more competitive with other solutions. These results again demonstrate the lightweight and efficiency of our proposed IDS when it is deployed at network gateways.

## 5. Limitations and Future Works

Although Realguard is a potential NIDS solution for IoT gateways, it still lacks full maturity to widely apply in commercial security systems. Its main disadvantages are listed below:

- RealGuard requires well-labeled traffic data to train the attack detection model. However, these data are uncommon, and building them requires a massive effort.
- RealGuard is potentially vulnerable to adversarial attacks due to missing barrier layers that mitigate the effect of adversarial samples.
- The attack detection model has to be frequently re-trained to maintain high accuracy. This consumes significant computation and network resources to deploy and update the model.

In future work, we will implement Realguard as a dedicated module of OpenWrt, a popular open operating system for IoT gateways. In addition, an unsupervised attack detection model for Realguard will be developed to deal with the lack of labeled datasets. We will also enhance the privacy of Realguard by employing federated learning that trains the attack detection model across multiple decentralized edge devices containing the training data. In short, our proposal opens up a great opportunity to deploy DNN-based NIDSs at the resource-constraint local network gateway. We hope Realguard and its resources (docker container, datasets) are beneficial to further research into intrusion detection systems.

## 6. Conclusions

In this paper, we introduce Realguard, an DNN-based intrusion detection system operated at an IoT network gateway to detect multiple cyber attacks. It achieves this goal by employing a lightweight feature extraction algorithm and an efficient attack detection model, which are based on a damped incremental statistic algorithm and a deep neural network model respectively. Through practical experiments, we show that Realguard could accurately detect different attacks (10 attack types) with a small computational footprint. In addition, it is efficient enough to run on a Raspberry PI in real time. 

## Figures and Tables

**Table 1:** The summary of related works on NIDS.

Year	Authors	Research Aspect	Model	Datasets	Num of Label	Data Analyzed	Performance
2017	Midi et al. [21]	Kalis: An IDS capable of detecting assaults in real-time across a broad variety of IoT systems	Signature based	Custom dataset	8	Packet-based	ACC = 100% TPR = 91%
2017	Liu et al. [22]	A CNN-base NIDS	CNN	KDD-Cup99	5	Flow-based	DR = 97.66% FAR = 0.1%
2018	Mirsky et al. [23]	An Ensemble of Autoencoders for real-time NIDS	ANN	Custom dataset	10	Packet-based	TPR = 99.99%, AUC = 99.99%
2019	Ahmim et al. [24]	NIDS that incorporate diverse classifier methodologies	REP Tree, JRIP, RF	CICIDS2017	15	Flow-based	DR = 94.475% ACC = 96.66% FAR = 1.145%
2019	Faker et al. [25]	Intrusion detection on ML/DL methodologies	DNN; RF; Gradient Boosting	CICIDS2017 UNSW UB15	14 9	Flow-based	ACC= 91–98%
2020	Wang et al. [26]	A deep hierarchical model for detecting anomaly traffic at packet-level	CNN-LSTM; CNN-GRU	ISCX2012 USTC-TFC2016 CICIDS2017	5 10 6	Packet-based	ACC = 99–100% DR = 99–100%
2020	Sun et al. [27]	A hybrid model of CNN and LSTM to extract network features and enhance NIDS	CNN + LSTM	CICIDS2017	7	Flow-based	ACC = 98.67% TPR = 97.21% FPR = 0.47%
2020	Mohammadpour et al. [28]	New CNN architecture for detecting particular abnormality	CNN	CICIDS2017	11	Flow-based	ACC = 99.46% FPR = 0.23% PPV = 99.76%
2020	Kaiyuan et al. [29]	A NIDS incorporated hybrid sampling and a deep hierarchical network	CNN + BiLSTM	NSL-KDD UNSW-NB15	5 10	Flow-based	ACC = 76–82%
	Our	Realguard: Realtime IDS for IoT Gateway	DNN	CICIDS2017	11	Packet-based	ACC = 99.93% TPR = 99.57% FPR = 0.04%

**Table 2:** The environments used to evaluate Realguard.

		Edge Gateway Raspberry PI 4B	Edge Server PC
CPU	Type	Broadcom BCM2711	Intel i7-9750H
	Clock	1.5 GHz	2.60 GHz
	Cores	Quad core Cortex-A72 × 4	4 (8 logical)
RAM		8 GB	16 GB

**Table 3:** Details of the evaluated datasets.

Attack Type	Description	Total Packet	Used Packet
Normal	Normal connection	11,926,723	400,000
FTP-Patator	File transfer protocol—brute force attack	110,736	110,736
SSH-Patator	Secure shell protocol—brute force attack	136,073	136,073
DoS Slowloris	Attackers flood the victim machine with malicious requests to overload victim systems	47,596	47,596
DoS Slowhttptest	Attackers flood the victim machine with malicious requests to overload victim systems	39,254	39,254
DoS Hulk	Attackers flood the victim machine with malicious requests to overload victim systems	2,245,526	200,000
DoS GoldenEye	Attackers flood the victim machine with malicious requests to overload victim systems	106,177	106,177
Heartbleed Port 444	Exploited by sending a malformed heartbeat request with a small payload and large length field to the vulnerable party	47,551	47,551
Botnet ARES	Zombie machine controlled by bot onwer, can be used to perform various attacks	9871	9871
DDoS LOIT	Distributed Denial of Service is an attempt to make victim services down by using multiple sources. This can be done by using botnet	1,280,602	200,000
Port Scan	Specify which port is opening for a particular service. Attacker use this to get information.	327,253	200,000

**Table 4:** Comparing binary detection performance between Realguard and its competitors.

(%)	Realguard	NB-SVM	DT-EnSVM	DBN	PSO+LSTM-RNN	PSO+DNN	XGB	AE+ANN
**TPR**	**99.66**	99.46	99.15	99.00	98.68	97.58	97.40	95.81
**FPR**	0.40	3.00	4.00	2.10	**0.16**	0.28	12.00	1.23
**ACC**	**99.64**	98.92	98.46	98.24	98.83	97.85	91.36	98.18

**Table 5:** Details of comparing multi-class attack detection quality between Realguard and its competitors.

(%)	Realguard	MLP	CNN-MCL	XGB	RF	SVC	ANN	LSTM	E-ML	REP Tree
**TPR Normal**	99.60	99.66	x	99.85	**99.93**	98.89	99.73	99.69	x	x
**TPR Botnet**	**98.46**	91.39	95.19	x	64.45	79.18	38.36	35.81	46.47	47.76
**TPR DoSSlowhttptest**	99.48	**99.75**	91.50	94.45	99.36	83.65	98.82	98.64	93.84	75.36
**TPR DoSGoldenEye**	**99.98**	**99.98**	98.71	99.27	99.76	99.91	99.08	97.62	67.57	66.43
**TPR DoSslowloris**	99.52	**99.85**	97.96	91.62	99.14	98.04	98.27	97.07	97.76	92.73
**TPR DoSHulk**	98.45	97.54	99.10	**99.92**	99.85	93.36	99.73	99.02	96.78	92.22
**TPR FTP-Patator**	99.92	**99.99**	99.77	x	99.94	99.95	99.62	99.68	99.64	99.18
**TPR SSH-Patator**	99.92	99.95	98.16	x	99.75	99.42	98.30	96.61	99.91	**100.00**
**TPR Heartbleed**	99.98	99.99	x	**100.00**	x	99.97	x	x	**100.00**	**100.00**
**TPR DDoS**	**100.00**	**100.00**	99.19	x	99.94	99.98	99.91	99.88	99.88	99.79
**TPR PortScan**	99.94	99.28	99.86	x	**99.95**	99.39	99.81	99.92	99.88	99.88
TPR (Avg)	**99.57**	98.85	97.72	97.52	96.21	95.61	93.16	92.39	90.17	87.33
FPR (Overall)	**0.04**	0.06	0.23	0.24	0.24	0.20	0.63	0.79	1.15	4.84
ACC (Overall)	**99.93**	99.89	99.46	99.54	99.86	99.64	99.58	99.57	96.67	93.40

**Table 6:** Comparing runtime performance between Realguard and its competitors.

		Train Rate (pkg/s)	Exec Rate (pkg/s)
Our	PC	6000	88,200
	Ras	1150	10,600
Kitsune [23]	PC	1100	37,300
	Ras	x	5400
Ahmim et al. [24]	PC	200	17,600
	Ras	x	x

**Table 7:** Compare resource consumption on Rasp Pi between Realguard and others.

	Realguard	Kitsune	RF	LSTM
**CPU (%)**	36.0	**33.8**	76.8	47.6
**RAM (MB)**	**114.5**	156.3	180.3	143.1

## Data Availability

Data available on request.
